# No difference in factual or conceptual recall comprehension for tablet, laptop, and handwritten note-taking by medical students in the United States: a survey-based observational study

**DOI:** 10.3352/jeehp.2022.19.8

**Published:** 2022-04-26

**Authors:** Warren Wiechmann, Robert Edwards, Cheyenne Low, Alisa Wray, Megan Boysen-Osborn, Shannon Toohey

**Affiliations:** 1 Departments of Emergency Medicine, University of California Irvine, Orange, CA, USA; 2 Department of Pathology and Lab Medicine, University of California Irvine, Orange, CA, USA; Hallym University, Korea

**Keywords:** Cognition, Digital technology, Handwriting, Medical students, Smartphone

## Abstract

**Purpose:**

Technological advances are changing how students approach learning. The traditional note-taking methods of longhand writing have been supplemented and replaced by tablets, smartphones, and laptop note-taking. It has been theorized that writing notes by hand requires more complex cognitive processes and may lead to better retention. However, few studies have investigated the use of tablet-based note-taking, which allows the incorporation of typing, drawing, highlights, and media. We therefore sought to confirm the hypothesis that tablet-based note-taking would lead to equivalent or better recall as compared to written note-taking.

**Methods:**

We allocated 68 students into longhand, laptop, or tablet note-taking groups, and they watched and took notes on a presentation on which they were assessed for factual and conceptual recall. A second short distractor video was shown, followed by a 30-minute assessment at the University of California, Irvine campus, over a single day period in August 2018. Notes were analyzed for content, supplemental drawings, and other media sources.

**Results:**

No significant difference was found in the factual or conceptual recall scores for tablet, laptop, and handwritten note-taking (P=0.61). The median word count was 131.5 for tablets, 121.0 for handwriting, and 297.0 for laptops (P=0.01). The tablet group had the highest presence of drawing, highlighting, and other media/tools.

**Conclusion:**

In light of conflicting research regarding the best note-taking method, our study showed that longhand note-taking is not superior to tablet or laptop note-taking. This suggests students should be encouraged to pick the note-taking method that appeals most to them. In the future, traditional note-taking may be replaced or supplemented with digital technologies that provide similar efficacy with more convenience.

## Introduction

### Background/rationale

Technological advances have radically transformed the tools that students use to learn. Laptops, handheld tablets, and smartphones are now everyday adjuncts to learning. In lieu of pen and paper, many students use handheld tablets for note-taking, often paired with a stylus, providing students with the ability to handwrite notes, draw pictures, and add other annotations directly onto digital documents. These technologies may enable learners to process information in innovative and comprehensive ways [1].

It has previously been hypothesized that longhand writing engages more complex cognitive processes than taking notes by typing. Past studies have reported that laptop typewritten notes tend to be verbatim note-taking that does not engage active learning. The most well-known study on the topic, by Mueller and Oppenheimer [2], found that longhand note-takers had stronger conceptual understanding and performance than laptop note-takers. As this study pre-dated the widespread use of tablet devices, it did not specifically address note-taking on a tablet with a touch or stylus input. In a study of brain activity using a stylus on a tablet, optimal conditions for learning were found with drawing on the tablet, indicating that the combination of tablet content visualization and handwriting may be beneficial for learning [3].

Surveys have shown that students prefer learning environments that incorporate technology; students expect higher education institutions to provide blended learning. Students choose to use certain technological devices to meet their own personalized learning needs [4]. Tablets are unique in their potential for software applications that provide note-taking tools, e-books, flashcards, videos, images, and practice tests [5]. Studies have shown an increase in performance with the use of tablet-based applications designed to teach anatomy [6]. However, research on the benefits of tablet-based note-taking is scarce.

### Objectives

The purpose of this study is to determine the effectiveness of digital note-taking strategies on learning comprehension and retention. We sought to re-create part of the study of Mueller and Oppenheimer [2] to evaluate tablet note-taking among a group of students who were comfortable with and commonly used this style of note-taking. We hypothesized that this new tablet-based note-taking would lead to equivalent or better recall as compared to written note-taking.

## Methods

### Ethics statement

The study was determined to be exempt by the Institutional Review Board of the University of California Irvine. We recruited students via email to voluntarily take part in the study. On registration for the study, they completed demographic questions regarding their educational background, knowledge of the topic, and preferred note-taking method. Obtaining informed consent from the participants was also exempted.

### Study design

This was a survey-based observational study that utilized part of the design of the study of Mueller and Oppenheimer [2] to investigate the efficacy of digital note-taking strategies on learning processes and understanding of concepts.

### Setting

We used the first of 3 experiments in the study of Mueller and Oppenheimer [2] as a framework for our study, which examined the effect of note-taking on a laptop versus handwriting on academic performance [3]. Demographic information was obtained via email when students registered to complete the study (Supplement 1). In August 2018 at the University of California Irvine on the day of the study, participants gathered in a lecture hall. The students were divided into groups of longhand, laptop, and tablet note-taking based on their note-taking preferences. We felt that the most accurate representation of note-taking efficacy would be achieved by having students use the format with which they were most comfortable. The tablet group was encouraged to use their preference of typing, stylus, or a note-taking application.

The students watched a TED Talk by Kevin Slavin—“How algorithms shape our world” (https://ed.ted.com/lessons/kevin-slavin-how-algorithms-shape-our-world) and were advised to take notes using their chosen method in preparation for a quiz afterward. This video was selected because it was one of the videos used in the original study by Mueller and Oppenheimer [2], and its topic was unfamiliar to students. Unlike the varied videos and quizzes in the study of Mueller and Oppenheimer [2], we used only 1 video and quiz for consistency. The video was played in a lecture hall. We encouraged participants to put away cell phones, stay silent during the study, and avoid surfing the internet or engaging in other tasks while the video was playing. After the video, students were asked to turn in notes by e-mail or paper copy to the study coordinator. Following this intervention, students watched a different TED talk video: “The art of Misdirection” by Apollo Robbins (https://www.ted.com/talks/apollo_robbins_the_art_of_misdirection). During this second video, students were advised not to take notes and to simply watch the video without any distractions. The purpose of the second video was to provide a wash-out period and act as a distractor between watching the first video and completing the quiz.

After the completion of the second video, students were given a 10-question short answer assessment about factual and conceptual concepts covered in the “How algorithms shape our world” video (Supplement 2). Students were given 30 minutes to complete the quiz. The quiz was identical to the quiz used in the study of Mueller and Oppenheimer [2] for this video.

### Participants

There were 51 participants in this study and all were 1st- or 2nd-year medical students at the University of California, Irvine School of Medicine. We recruited students via email to voluntarily take part in the study. Upon registration for the study, they completed demographic questions including name, educational background (type of degree[s] and title of degree[s]), age (numeric entry), gender, (selection–male, female), and preferred note-taking method (selection–iPad/tablet, pen and paper, laptop).

### Variables

Quantitative scores of correct answers for each video measuring both factual and conceptual comprehension were the primary variable. Secondary variables included an analysis of the content of student notes, including word count, presence/absence of drawings, highlights, and other media/tools between conditions.

### Data source/measurement

This study used a quiz identical to that administered in the study of Mueller and Oppenheimer [2] for the “How algorithms shape our world” video. The questionnaire consisted of a 10-question short answer assessment about factual and conceptual concepts in the video. While the validity and reliability of the assessment were not measured, we recreated a previously published study and as such used the same videos and assessments. We did not complete additional validation or reliability evaluations for the assessment. Notes were scanned into PDF format, and the word count, presence/absence of drawings, highlights and other media/tools were evaluated manually by 2 independent reviewers with a third reviewer resolving any discrepancies.

### Bias

To minimize potential sources of bias, 2 blinded reviewers graded each quiz using the same grading scale as Mueller and Oppenheimer [2], assigning points for each factual and conceptual-based question. A third reviewer resolved any discrepancies in scores.

### Study size

Based on previous studies, we performed a power analysis utilizing the independent-samples Kruskal-Wallis test. With an 80% power, alpha of 0.05, and assessing a 20% difference in retention between groups, a sample size of at least 17 students was needed in each group. Of note, participants were allowed to self-select their preferred note-taking method to minimize bias from utilizing a less familiar method of note-taking.

### Statistical methods

Statistical analysis was performed using the 1-sample Kolmogorov-Smirnov test to examine whether the factual score and conceptual score followed a normal distribution. We reported the median and interquartile range (IQR), as the score distribution was not normal. We used the independent-samples Kruskal-Wallis test to compare the score distribution among the study groups. We considered a P-value less than 0.05 to be statistically significant. We used IBM SPSS ver. 26.0 for Windows (IBM Corp., Armonk, NY, USA) for data analysis.

## Results

### Participants

Sixty-eight out of 208 medical students participated in the study, of whom 31 were male and 37 were female. The participants ranged in age from 21 to 29 years old, with an average age of 24 years old.

### Main results

When asked which note-taking method between tablet, paper, and laptop is better for learning, 59.7% (40/67) participants stated that an iPad/tablet was better, while 40.3% (27/67) said that pen and paper note-taking was better. Furthermore, 68.7% (46/67) said their note-taking method of choice did not differ depending on the type of lecture. Of note, 1 participant did not answer this question (67/68). We asked participants about their preferred note-taking method and reasoning behind it; for their preferred note-taking method, 58 students selected iPad/tablets, 4 chose pen and paper, and 6 chose a combination of both methods. All 68 participants answered this question. Students who chose tablets highlighted the efficiency of consolidating notes in one place, the ease of annotating lecture slides, drawing diagrams, writing with the stylus, the accessibility of online study materials, syncing across devices, customizing/editing notes, and portability. Those who used pen and paper regularly emphasized how handwriting helps with memory and stated that writing content out in one’s own words reinforces concepts, minimizes distractions from technology, and allows students to better engage with the material.

Since students were allowed to self-select into groups depending on their preferred type of note-taking, the groups were slightly uneven, with 42 students in the tablet group, 19 students in the longhand group, and 7 students in the laptop group. While we did not reach the minimum number of students in the laptop group, which was a side effect of this method being uncommon in our population, we still reported these results.

Each student was asked to report how much knowledge related to the topic of the talk they had before the study on a numerical scale from 1, meaning “none at all,” to 5, meaning “expert.” Table 1 shows the distribution of the participants in the study groups and their expressed prior knowledge of the subject.

**Fig. 1.**
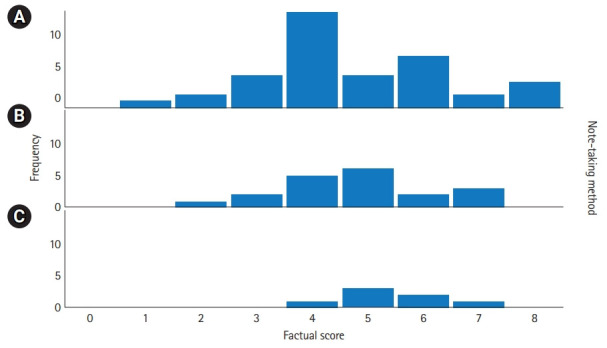
Distribution of factual scores. Note-taking method: (A) tablet, (B) longhand, and (C) laptop.

Fig. 1 shows the distribution of factual scores among the study groups. The median factual score was 4 (1st quartile: 4, 3rd quartile: 6) in group A, 5 (1st quartile: 4, 3rd quartile: 6) in group B, and 5 (1st quartile: 5, 3rd quartile: 6) in group C. There was no significant difference in the factual recall scores across the tablet, laptop, and handwritten note-taking conditions (P=0.38).

**Fig. 2.**
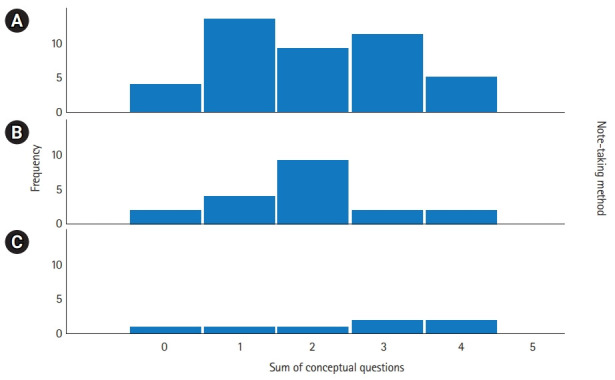
Distribution of conceptual scores. Note-taking method: (A) tablet, (B) longhand, and (C) laptop.

Fig. 2 shows the distribution of conceptual recall scores among the study groups. The median conceptual score was 2 (1st quartile: 1, 3rd quartile: 3) in group A, 2 (1st quartile: 1, 3rd quartile: 2) in group B, and 3 (1st quartile: 1, 3rd quartile: 4) in group C. There was no significant difference in the conceptual application scores across tablet, laptop, and handwritten note-taking (P=0.61).

The median word count was 131.5 (IQR, 95.0 to 175.0) for tablets, 121.0 (IQR, 96.0 to 159.0) for handwriting, and 297.0 (IQR, 216.0 to 358.0) for laptops (P=0.01). The total number of drawings, highlighting, or other media/tools was 17 (40.5%) in the tablet group, 3 (15.8%) in the handwriting group, and 0 (0.0%) in the laptop group (P=0.029) (Table 2).

Raw response files of students to a quiz were available from Dataset 1.

## Discussion

### Key results

There was no significant difference in the factual or conceptual recall scores for tablet, laptop, and handwritten note-taking. The tablet group had the highest presence of drawing, highlighting, and other media/tools.

### Interpretation

Results demonstrated that there was no significant difference between tablet devices, laptops, and handwriting in conceptual or factual comprehension, suggesting they are similar in efficacy. It is important to note that the sample size of the laptop students was extremely small and unlikely to generate statistically significant results. Therefore, tablet note-taking can be encouraged as an option for students on par with longhand notes.

### Comparison with previous studies

It has been established that taking notes is beneficial for learning by creating new neural connections that enforce memory. This is true for both lecture and textbook reading note-taking [7]. Students remember more when they take notes and can use that information for testing [8]. The act of taking notes may be more beneficial than the notes themselves. Note-taking is cognitively demanding because it requires listening intently to the lecture while reading the PowerPoint and writing organized and comprehensible notes [9]. Passively listening to a lecture does not require this effort. Laptop note-taking offers a faster method of note-taking, which may be beneficial in situations such as video or lecture speaking when students must multitask by hearing new information while simultaneously taking notes. Some researchers have found no difference in performance between laptop and paper note-taking, while Bui et al. showed benefits to laptop note-taking during audio lectures [10]. This contradicts the findings of Mueller and Oppenheimer [2], and it has been speculated this may be due to the more complex information given during a lecture compared to a TED Talk, when the speed of note-taking may give students more time to focus on the complicated material being presented [11].

Digital note-taking encompasses laptops, tablets, and note-taking applications. Across all platforms, the advantage of taking notes digitally are speed, ability to search, and legibility [12]. Tablets are unique due to the ability to draw, add color, type, handwrite, highlight text, and add images and links to study resources. In a study by Wammes et al. [9], drawing compared to writing was found to better enhance memory by integrating visual, motor, and semantic processes. Revising notes is quick and easy on a tablet, and studies have shown that students who edit notes will retain more information [13]. To best incorporate tablet devices, educators should provide lectures in PowerPoint form before class so they can be readily downloaded and annotated. Note-taking applications such as Notability, Evernote, Zoho Notebook, and OneNote are becoming popular tools with their feature-rich and syncing capabilities. Creative learning strategies such as mind mapping and sketching are already taking digital forms and can be incorporated into learning on a tablet [14]. Students and educators must be educated on digital note-taking strategies so they can best utilize and promote them in the classroom.

### Limitations

The limitations of this study include participant selection. The medical students recruited all had access to tablet devices, which may not be representative of students on average and may have introduced selection bias into our study. Each student chose their preferred method of note-taking instead of random sorting into groups. Confounding variables, such as previous knowledge of the topic, were not considered in the analysis. Additionally, the sample size was limited for the students who used laptops, so the groups were not evenly distributed. Although we calculated the power necessary for the study a priori, a significant difference might be found with a larger sample size.

### Generalizability

The University of California Irvine School of Medicine’s iMedEd initiative is a completely digital and interactive curriculum, using digital textbooks, podcasts, audio/video libraries, and online curriculum materials. Each first-year medical student receives an iPad accompanied by a dedicated “on-boarding” session that demonstrates how to use this device for note-taking. Through informal surveys, most medical students embrace the technology and use iPads as their sole note-taking method during medical school. This makes our study population of the University of California Irvine medical students distinctive for their proficiency and comfort using tablet devices. Although the minimum number of students was not reached in the laptop group, the results of this study demonstrate how other schools could benefit from incorporating a digital curriculum.

### Suggestions

As stated above, future studies with larger and equal sample sizes, along with better control of biases and confounding factors, should be performed to further determine the benefit of using tablet-based note-taking over handwritten or laptop notes.

### Conclusion

Most of the current research compares laptops and handwriting, while there is limited research exploring tablet note-taking capabilities. Although there is conflicting research regarding the best note-taking method, our study showed that longhand note-taking did not give a performance advantage when compared to tablets or laptops. Tablet devices provide a combination of digital functionality with the cognitive benefits of handwriting. As technology continues to be incorporated into the classroom, traditional notebooks can be replaced with digital technologies that provide the same advantages with more convenience.

## Figures and Tables

**Table 1.** Distribution of participants and prior knowledge

Note-taking method	Prior knowledge of the topic				
	Count	Min	Max	Median	Mean
Tablet	42	1	3	1	1
Pen	19	1	4	1	1
Laptop	7	1	3	1	1

A numerical scale from 1 to 5 was used with 1 being “none at all” and 5 being “expert.”

**Table 2.** Word count and use of other tools

Variable	Presence of							
	Word count		Drawing		Highlight		Other media	
	Median (IQR)	P-value	No. (%)	P-value	No. (%)	P-value	No. (%)	P-value
Note-taking method		0.001		0.373		0.089		0.089
Tablet (N=42)	131.5 (95.0 to 175.0)		3 (7.1)		7 (16.7)		7 (16.7)	
Pen (N=19)	121.0 (96.0 to 159.0)		3 (15.8)		0		0	
Laptop (N=7)	297.0 (216.0 to 358.0)		0		0		0	

IQR, interquartile range.

## Footnotes

**Data availability**
Data files are available from Harvard Dataverse: https://doi.org/10.7910/DVN/CD3NXD Dataset 1. Raw response files of students to a quiz identical to that administered in the study by Mueller and Oppenheimer [2] for the “How algorithms shape our world” video with a coding book.

## Supplementary materials

Data files are available from Harvard Dataverse: https://doi.org/10.7910/DVN/CD3NXD

Supplement 1. Demographic questions.

Supplement 2. Ten-item quiz identical to that used in Mueller and Oppenheimer’s study [2] for the “How algorithms shape our world” video.

Supplement 3. Audio recording of the abstract.

## References

[1] Mueller PA, Oppenheimer DM (2016). Technology and note-taking in the classroom, boardroom, hospital room, and courtroom. Trends Neurosci Educ.

[2] Mueller PA, Oppenheimer DM (2014). The pen is mightier than the keyboard: advantages of longhand over laptop note taking. Psychol Sci.

[3] van der Meer AL, van der Weel FR (2017). Only three fingers write, but the whole brain works: a high-density EEG study showing advantages of drawing over typing for learning. Front Psychol.

[4] Dahlstrom E, Walker JD, Dziuban C (2013). ECAR study of undergraduate students and information technology, 2013. ECAR study of undergraduate students and information technology.

[5] Piolat A, Olive T, Kellogg RT (2005). Cognitive effort during note taking. Appl Cogn Psychol.

[6] Bui DC, Myerson J (2014). The role of working memory abilities in lecture note-taking. Learn Individ Differ.

[7] Sana F, Weston T, Cepeda NJ (2013). Laptop multitasking hinders classroom learning for both users and nearby peers. Comput Educ.

[8] Stacy EM, Cain J (2015). Note-taking and handouts in the digital age. Am J Pharm Educ.

[9] Wammes JD, Meade ME, Fernandes MA (2016). The drawing effect: Evidence for reliable and robust memory benefits in free recall. Q J Exp Psychol (Hove).

[10] Kay R, Lauricella S (2011). Exploring the benefits and challenges of using laptop computers in higher education classrooms: a formative analysis. Can J Learn Technol.

[11] Bennett J, McKain D, Khan AA, Umair S (2018). Handbook of research on mobile devices and smart gadgets in K-12 education.

[12] Rosciano A (2015). The effectiveness of mind mapping as an active learning strategy among associate degree nursing students. Teach Learn Nurs.

[13] Mangen A, Anda LG, Oxborough GH, Brřnnick K (2015). Handwriting versus keyboard writing: effect on word recall. J Writ Res.

[14] Luo L, Kiewra KA, Flanigan AE, Peteranetz MS (2018). Laptop versus longhand note taking: effects on lecture notes and achievement. Instr Sci.

