# Supplementary material for: No difference in factual or conceptual recall comprehension for tablet, laptop, and handwritten note-taking by medical students in the United States: a survey-based observational study
Source: J Educ Eval Health Prof. 2022 Apr 26;19:8. doi: 10.3352/jeehp.2022.19.8 (PMC9247713; doi:10.3352/jeehp.2022.19.8)
Supplement: Supplementary file 2 — Supplement 1. Demographic questions. [file jeehp-19-08-suppl1.docx]

**Supplement 1.** Demographic questions.

1. Name:

2. Educational background (type of degree[s] and title of degree[s]):

3. Age: (numeric entry)

4. Gender: (selection–male, female)

5. Preferred note taking method: (selection–iPad/tablet, pen & paper, laptop)
