# Supplementary material for: No difference in factual or conceptual recall comprehension for tablet, laptop, and handwritten note-taking by medical students in the United States: a survey-based observational study
Source: J Educ Eval Health Prof. 2022 Apr 26;19:8. doi: 10.3352/jeehp.2022.19.8 (PMC9247713; doi:10.3352/jeehp.2022.19.8)
Supplement: Supplementary file 3 — Supplement 2. Ten-item quiz identical to that used in Mueller and Oppenheimer’s study [2] for the “How algorithms shape our world” video. [file jeehp-19-08-suppl2.docx]

| **Coding book** |
| --- |
|  |
| Q1 |
| 2 = complete answer about being fast and about being able to find patterns humans cannot |
| 1 = one of the pieces (either fast or finding patterns) |
| 0 = anything else |
| (Index score: 1, 0.5, 0) |
| Q2 |
| 1 = 70% |
| 0 = anything else |
| (Index score: 1, 0) |
| Q3 |
| 2 = good answer about human control and algorithms being locked in loops |
| 1 = something about a lack of human control |
| 0 = anything else |
| (Index score: 1, 0.5, 0) |
| Q4 |
| 1 = Pragmatic Chaos |
| 0 = anything else |
| (Index score: 1, 0) |
| Q5 |
| 2 = complete answer about algorithms changing our actual landscape, as in the picture |
| 1 = partial answer about it being a metaphor |
| 0 = something not about algorithms modifying our actual landscape |
| (Index score: 1, 0.5, 0) |
| Q6 |
| 2 = NYC and Chicago |
| 1 = one of these |
| 0 = anything else |
| (Index score: 1, 0.5, 0) |
| Q7 |
| 1 = 4 |
| 0 = anything else |
| (Index score: 1, 0) |
| Q8 |
| 1 = answer about predicting success of a movie (based on a script) |
| 0 = other answers |
| (Index score: 1, 0) |
| Q9 |
| 1 = Nanex |
| 0 = anything else |
| (Index score: 1, 0) |
| Q10 |
| 2 = The Carrier Hotel |
| 1 = a hotel |
| 0 = anything that isn’t a hotel |
| (Index score: 1, 0.5, 0) |
